# Case report of right hamate hook fracture in a patient with previous fracture history of left hamate hook: is it hamate bipartite?

**DOI:** 10.1186/1746-1340-14-22

**Published:** 2006-10-12

**Authors:** Marion W Evans, Micheal L Gilbert, Sandra Norton

**Affiliations:** 1 Parker College of Chiropractic Research Institute, 2500 Walnut Hill Lane, Dallas, TX 75229, USA; 2 Resident, Parker College of Chiropractic Department of Radiology, 2500 Walnut Hill Lane, Dallas, TX 75229, USA; 3 Chair – Parker College of Chiropractic Department of Radiology, 2500 Walnut Hill Lane, Dallas, TX 75229, USA

## Abstract

**Background:**

Hamate hook fracture is a common fracture in golfers and others who play sports that involve rackets or sticks such as tennis or hockey. This patient had a previous hamate fracture in the opposing wrist along with potential features of hamate bipartite.

**Case presentation:**

A 19 year old male presented with a complaint of right wrist pain on the ulnar side of the wrist with no apparent mechanism of injury. The pain came on gradually one week before being seen in the office and he reported no prior care for the complaint. His history includes traumatic left hamate hook fracture with surgical excision.

**Conclusion:**

The patient was found to have marked tenderness over the hamate and with a prior fracture to the other wrist, computed tomography of the wrist was ordered revealing a fracture to the hamate hook in the right wrist. He was referred for surgical evaluation and the hook of the hamate was excised. Post-surgically, the patient was able to return to normal activity within eight weeks. This case is indicative of fracture rather than hamate bipartite. This fracture should be considered in a case of ulnar sided wrist pain where marked tenderness is noted over the hamate, especially after participation in club or racket sports.

## Background

Wrist pain is often seen in chiropractic practices [1]. While fracture to the scaphoid or navicular is the most prevalent of wrist fractures [2], hamate hook fracture is the most frequent fracture in golfers [3]. In most cases, the lead wrist, which is the left wrist in a right handed golfer, is most commonly fractured when the player strikes the ground, root or rock prior to striking the ball. This leads to twisting of the butt of the club against the hamate hook resulting in a fracture, typically of the lead wrist which is the left wrist in a right-handed golfer [3].

Occasionally, conservative care heals the fracture [4]. However, in many cases the hook must be surgically removed before normal function will be restored without pain [5]. Commonly, the diagnosis is delayed due to initial radiographs being read as negative, a more prominent injury being seen at the time of initial presentation or the stoic nature of the athlete who may delay evaluation [6].

## Case presentation

**Figure 1:**
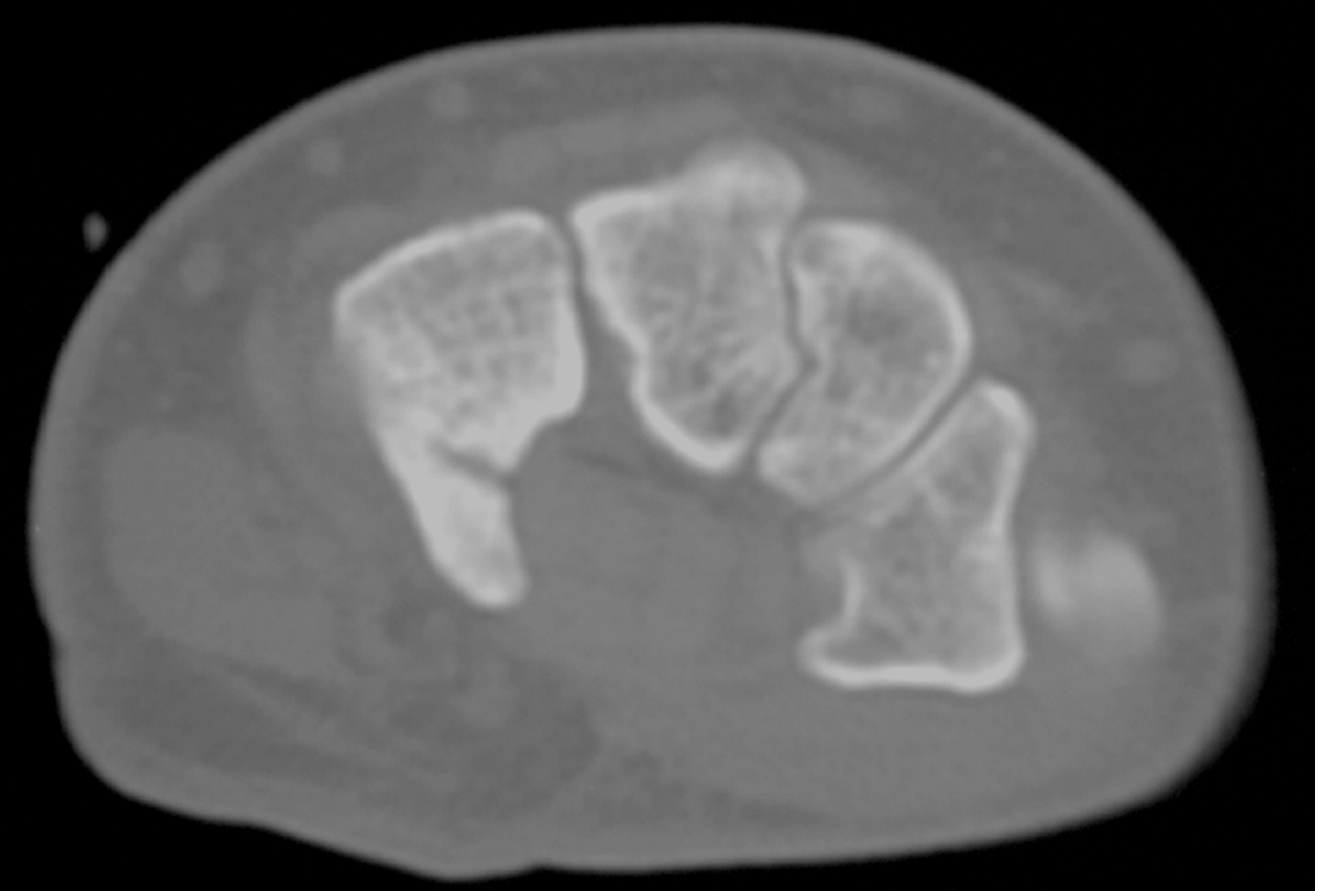
CT of left wrist indicating hamate hook fracture.

The patient was a 19 year old male who was 204.2 cm in height and weighted 145.15 kg. He was afebrile and had a blood pressure of 128/80, left arm, seated. Otherwise healthy, he experienced gradual right wrist pain over the hamate and did not report a traumatic golf injury, although he does play golf. He had a previous fracture to the left hamulus over one year prior [Fig 1] that apparently occurred on an attempt at ball strike with a sand wedge while playing golf, in which he struck a rock just behind the ball. In that case, immediate pain was noted and the condition was misdiagnosed by a sports medicine clinic prior to evaluation by the chiropractic clinician in his chiropractic office [7]. The left hook had to be excised due to failure of fragment fusion after plaster splinting, which was applied for six weeks for the treatment of a suspected scaphoid fracture. The patient's wrist injury healed post-surgically with some complications involving a subsequent navicular-lunate ligament tear and the patient was eventually able to return to golf.

Since there was a previous misdiagnosed fracture to the left wrist in this case, the patient called the chiropractic office first. Due to his history, computed tomography [CT] was ordered immediately following an examination which demonstrated mild to moderate pain on all right wrist movements, point tenderness over the hamate and previous difficulty in obtaining a carpal tunnel view on plain film x-ray.

**Figure 2:**
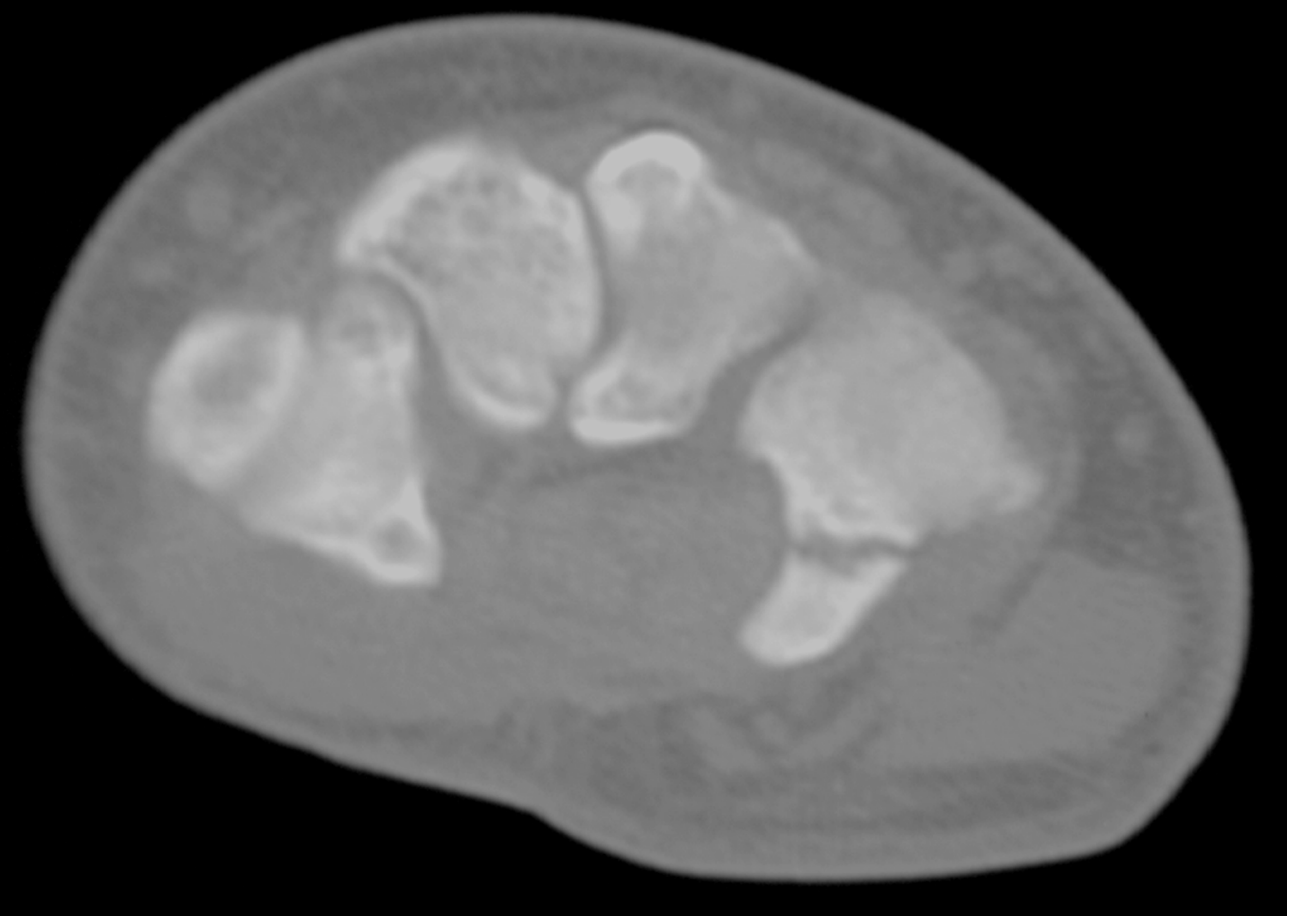
CT of right wrist indicating hamate hook fracture.

The CT scan revealed a complete, slightly displaced fracture of the hook of the right hamate with associated soft tissue edema [Fig 2]. A referral to an orthopedic surgeon was made to assess the need for excision of the hook of the hamate. Because of prior history, the patient elected to have the hook excised without conservative therapy.

Bilateral fracture of the hamate is uncommon. In a case-report by Bray, Swafford and Brown in 1985 [8], their search of the literature found only 19 cases prior to 1977. In this case, the left wrist had an apparent mechanism of injury classic for fracture at this site, as it is the left hamate that contacts the butt of the club in a right-handed golfer [3]. However, the right wrist did not have this mechanism of injury. This may suggest some preexisting condition of the hamate in this patient. A condition known as hamate bipartite affects the hamate in some patients [9,10]. This condition, which is thought to be the result of fibrocartilagenous union between the body and hook of the hamate, typically causes symptoms in this part of the wrist and is characterized by a weak or ununited appearance of the hook that can be detected on CT or plain film radiographs [10].

Hamate bipartite tends to be suspected in cases where there is no history of trauma or surgery to the wrist [10]. In our case, there was a denial of traumatic golf injury to the right wrist but was apparent in the left.

### Features of hamate bipartite

Features of hamate bipartite according to Pierre-Jerome & Roug [10] include;

• Bilaterally similar bipartite hamulus

• No sign or history of traumatic wrist injury or edema or soft tissue changes suggestive of un-united fracture

• Equal size and uniform signal intensity on MRI evaluation of each part

• Absence of progressive degenerative changes between the two components of the hamate or elsewhere in the wrist

• Smooth, well corticated and rounded margins of the hamate and un-united hook.

Symptoms were noted in the case of the left fracture immediately upon the patient's dubbed ball strike and only surgical excision relieved the pain [7]. Additionally, the original radiological report accompanying the images of the right wrist was indicative of fracture and not otherwise. The attending radiologist noted degenerative changes within the right wrist, separation of the naviculolunate interspace and 1–2 mm of separation of the hook from the body of the hamate. There was also some indication of a possible previous fracture of the capitate noted by the radiologist, which would further indicate possible previous trauma to the right wrist. However, the patient denied previous trauma.

### Diagnostic Imaging Considerations

Hamate fractures represent approximately 2–4% of all fractures involving the carpal bones [11]. Fractures involving the hamulus, or hook, represent one of the two groups of fractures of the hamate [12]. Norman and others documented three radiographic signs suggestive of fracture of the hamulus [13]. According to their criteria, the most frequently encountered and most important feature is the lack of visualization of the hook. On the dorsovolar view the hamulus is seen en face super-imposed over the hamate and demonstrates a cortical ring shadow known as the "eye sign" [12]. A blurry or indistinguishable appearance of the "eye", as well as sclerosis of the hamulus, seen associated with nonunion, represents the other two radiographic features that suggest fracture of the hook [12,13].

Various radiographic positioning techniques can prove useful in the evaluation of potential hamate fractures. These may not always be of diagnostic quality due to limited range of motion experienced by the patient as a result of pain, especially in acute or subacute fractures [12]. Moreover, these fractures are commonly overlooked on standard radiographic studies of the wrist due to the lack of specific physical exam findings and a low index of suspicion [14]. Conventional radiographic examination of the wrist usually consists of the dorsovolar view, which demonstrates the radiographic signs first described by Norman and colleagues as discussed previously, as well as the lateral and medial oblique projections [15].

**Figure 3:**
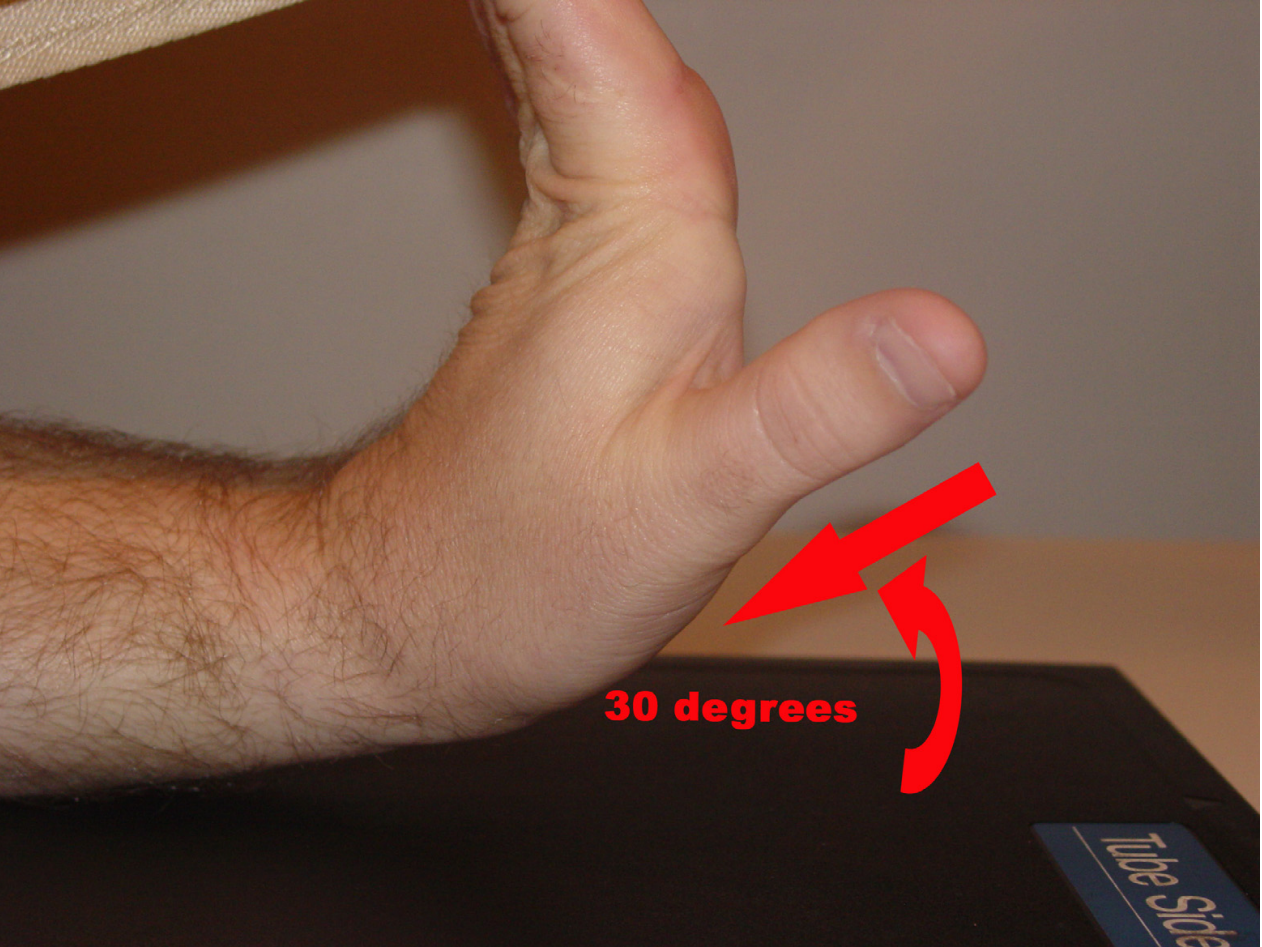
Radiographic position for carpal tunnel view.

The use of the carpal tunnel view [Fig 3] has increased in an attempt to better elucidate the presence of a hamulus fracture [15]. Originally described by Gaynor and Hart [13,15], it is set with the patient positioned such that the flexor surface of their forearm lies against the film with slight radial rotation and the long axis of their hand is made as vertical as possible. The central ray is directed toward the palmar surface distal to the base of the third metacarpal with 25–30° of tube angulation. The patient may use their other hand or some other appropriate device to hold their wrist in this extended position [16].

**Figure 4:**
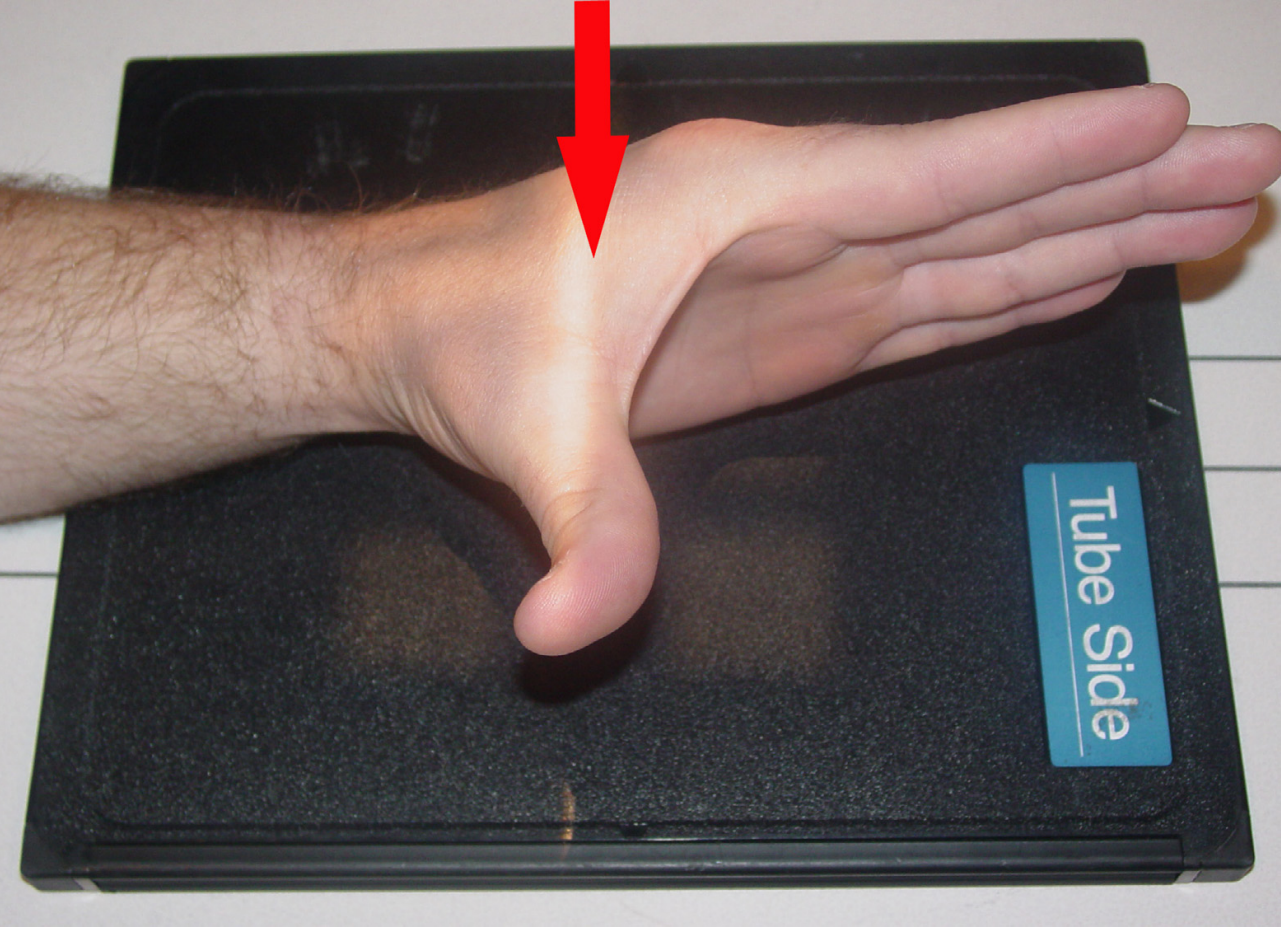
Radiographic position for radial-deviated, thumb-abducted view.

The "radial-deviated, thumb-abducted lateral view" [Fig 4] is considered by some authors, an underused technique that adequately demonstrates the hamate between the thumb and index finger and clearly displays fractures of the hamulus [15]. This radiograph is performed by positioning the patient with their forearm in neutral and the medial aspect of their wrist against the film cassette. Their thumb is fully extended and abducted and their wrist deviated radially. This position results in maximum widening of the index finger-thumb web space [15]. The central ray is directed at the center portion of the index finger and thumb web. Alternately, the radial deviation can be excluded and a 15° tube angulation, oriented toward the wrist, can be used [15]. Bhalla and colleagues consider this view a cost-effective and time-saving adjunct to traditional wrist series when fracture of the hook of the hamate is suspected.

The hamulus, which develops from its own ossification center, may fail to fuse with the body of the hamate [17]. This normal variant is referred to as the os hamuli proprium and may be difficult to differentiate from avulsion fractures of the hamate hook [17]. In equivocal cases, computed tomography [CT] of the wrist is an effective advanced imaging technique to confirm the diagnosis of hamulus fractures due to its ability to provide an image that is orthogonal to the plane of the hamulus base fracture, while avoiding the possibility of superimposed anatomical structures [12,15]. In fact, it has been suggested that it is pointless to obtain plain films when hamulus fracture is suspected clinically and that CT should be the initial imaging modality chosen [18].

Moreover, with the newer generation spiral CT multiple imaging planes can be obtained after a single scan [18]. With complete fractures, CT clearly reveals an osseous fragment demonstrating indistinct and irregular apposing cortical margins separated from the parent bone [14]. Incomplete fractures exhibit partial cortical disruption without osseous fragment separation. Additionally, internal joint derangements, such as injuries to the triangular fibrocartilage complex, may be found in association with fracture of the hamulus depending on the mechanism of injury such as a fall on the outstretched, pronated arm. Physical examination of these patients reveals tenderness between the pisiform and ulnar styloid on the ulnar border of the wrist [19]. Typically, however, there would be no indication of fracture in these patients and imaging would make the differential diagnosis [20]. Further, magnetic resonance imaging [MRI] is, in the opinion of the authors, most effective in determining the presence and extent of these injuries.

## Conclusion

We propose that in spite of no known mechanism of injury to the right wrist in the patient, the left wrist was traumatically fractured, as he felt immediate pain that completely resolved after surgical excision of the hook. We also suggest one other possibility in this patient. This is a young man who is very large for his age. His height and anthropometric features would suggest that his wrist bones are very large as well. This could make the hook of the hamate longer and therefore, weaker where the hook extends from the body of the hamate. Perhaps this made his bone more vulnerable to fracture. Perhaps, in spite of the patient's denial of traumatic injury, the fracture is related to his golf playing, as he is an avid player who spends quite a bit of time on the course. Further, the corresponding author has observed the swing of the young man while playing golf and he has a powerful swing as one might imagine in someone his size. The forces exerted on the wrist would speculatively, be above average.

While a case of hamate bipartite may difficult to rule out in some cases, it is rather curious to us that one wrist was apparently fractured while playing golf and the other not, approximately one year apart. We conclude that this is a case of bilateral fracture of the hamate, although they clearly occurred in separate events. Pain on the ulnar side of the wrist in those who participate in racket or club sports should be evaluated for fracture and hamate hook fracture should be given diagnostic consideration. Hamate bipartite should be considered in case of persistent pain where no prior history of trauma is noted.

## List of abbreviations

CT-computed tomography, MRI-magnetic resonance imaging

## Competing interests

The authors declare that they have no competing interests.

## Authors' contributions

ME treated the case and contributed to the sequence alignment and drafted the primary manuscript. MG contributed to the sequence alignment of the manuscript and coordinated additional material on diagnostic imaging considerations. SN contributed to the sequence alignment of the manuscript. All authors read and approved the final manuscript.
